# Hypoxic Preconditioning Suppresses Glial Activation and Neuroinflammation in Neonatal Brain Insults

**DOI:** 10.1155/2015/632592

**Published:** 2015-07-27

**Authors:** Chien-Yi Chen, Wei-Zen Sun, Kai-Hsiang Kang, Hung-Chieh Chou, Po-Nien Tsao, Wu-Shiun Hsieh, Wen-Mei Fu

**Affiliations:** ^1^Graduate Institute of Clinical Medicine, National Taiwan University College of Medicine, Taipei, Taiwan; ^2^Departments of Pediatrics, National Taiwan University Hospital and National Taiwan University College of Medicine, Taipei, Taiwan; ^3^Department of Anesthesiology, National Taiwan University Hospital and National Taiwan University College of Medicine, Taipei, Taiwan; ^4^Department of Pharmacology, College of Medicine, National Taiwan University, No. 1 Jen Ai Road, Section 1, Taipei 10051, Taiwan

## Abstract

Perinatal insults and subsequent neuroinflammation are the major mechanisms of neonatal brain injury, but there have been only scarce reports on the associations between hypoxic preconditioning and glial activation. Here we use neonatal hypoxia-ischemia brain injury model in 7-day-old rats and *in vitro* hypoxia model with primary mixed glial culture and the BV-2 microglial cell line to assess the effects of hypoxia and hypoxic preconditioning on glial activation. Hypoxia-ischemia brain insult induced significant brain weight reduction, profound cell loss, and reactive gliosis in the damaged hemisphere. Hypoxic preconditioning significantly attenuated glial activation and resulted in robust neuroprotection. As early as 2 h after the hypoxia-ischemia insult, proinflammatory gene upregulation was suppressed in the hypoxic preconditioning group. *In vitro* experiments showed that exposure to 0.5% oxygen for 4 h induced a glial inflammatory response. Exposure to brief hypoxia (0.5 h) 24 h before the hypoxic insult significantly ameliorated this response. In conclusion, hypoxic preconditioning confers strong neuroprotection, possibly through suppression of glial activation and subsequent inflammatory responses after hypoxia-ischemia insults in neonatal rats. This might therefore be a promising therapeutic approach for rescuing neonatal brain injury.

## 1. Introduction

Hypoxia-ischemia injury is the final common mechanism in different kinds of brain damage resulting from trauma. Hypoxia-ischemia injury is also the major cause of brain damage in neonates [1]. The incidence of hypoxia-ischemia encephalopathy is about 2.5 per 1,000 live births, and significant neurologic morbidity occurs in as many as 50–75% of these infants [2]. Long-term consequences, including cerebral palsy, cognitive deficits, seizure disorders, intellectual deficits, and behavioral problems, are lifelong and devastating [3]. To date, it is still a big challenge to protect the newborn brain from hypoxia-ischemia injury.

Pathophysiological features of cerebral hypoxia-ischemia are unique to the immature brain and provide the potential for clinical intervention [4]. Multiple mechanisms are involved in the pathogenesis of neonatal hypoxia-ischemia brain injury, including oxidative stress, apoptosis, and excitotoxicity. There is growing evidence to demonstrate the pivotal role of inflammation [1]. The inflammatory cascade is characterized by a rapid activation of resident microglial cells and infiltration of peripheral leukocytes into the injured parenchyma [5–7]. Astroglial cells are also activated and play a significant and sustained role in inflammation following brain trauma and hypoxia-ischemia. Once activated, these inflammatory cells contribute to neuronal damage through the release of cytotoxic mediators, including cytokines, chemokines, reactive oxygen species, adhesion molecules, and matrix metalloproteins [8]. Therapeutic approaches targeting anti-inflammation can diminish brain injury in animal models of neonatal insults, such as antineutrophil serum [9], IL-1 receptor antagonists [10], and the microglial inhibitor minocycline [11].

Hypoxic preconditioning, or hypoxia-induced tolerance, refers to a brief period of hypoxia that protects against an otherwise lethal insult occurring minutes, hours, or days later [12]. Gidday et al. [13] were the first to show the neuroprotective effect of hypoxic preconditioning in neonatal brain injury. They found a dramatic neuroprotective effect when 7-day-old rats were exposed to 8% oxygen (hypoxic admixture) for 3 hours, followed by combined hypoxia-ischemia (8% oxygen plus carotid artery occlusion) 24 hours later. Several subsequent studies further examined the underlying mechanisms, including the roles of the adenosine A1 receptor [14], vascular endothelial growth factor (VEGF) [15], erythropoietin (EPO) [16], nitric oxide synthase (NOS) [17], hypoxia-inducible factor (HIF) [18], glycogen [19], and others [20]. Most of these studies focused on neuronal [21] and vascular structure [22], but the role of glial cells in hypoxic preconditioning was seldom explored. The beneficial effects of hypoxic preconditioning might involve counterregulation of glial activation and subsequent inflammatory processes following the cerebral hypoxia-ischemia insult. In this study, we examined whether hypoxic preconditioning renders glial cells hyporesponsive due to hypoxia-ischemia induced activation. The anti-inflammatory effect of hypoxic preconditioning in glial cells was studied both* in vivo* and* in vitro*.

## 2. Materials and Methods

### 2.1. Animals

All experiments were performed in accordance with the National Institutes of Health Guidelines on Laboratory Animal Welfare and were approved by the Animal Care and Use Committee of the College of Medicine, National Taiwan University. Ten to twelve Sprague-Dawley pups per dam were used in this study and housed in institutional standard cages on a 12-hour light/12-hour dark cycle, with* ad libitum* access to water and food. The pups were housed with their dams until weaning on postnatal day 21 (P21). Only male rat pups were used in this study.

### 2.2. Hypoxic Preconditioning in Rat Pups

A modified Rice-Vannucci model was used for the induction of hypoxia-ischemia (HI) brain damage in 7-day-old (P7) neonatal rats [23]. P7 rats were anesthetized with isoflurane (3.5% for induction and 1.5% for maintenance) in a mixture of nitrogen and oxygen, delivered via a facial mask. The right common carotid artery was dissected and cut between double ligatures of Prolene sutures (6-0). After anesthesia and surgery, the animals were allowed to recover for at least 1 h. The litters were then placed in a 3.0-liter airtight plastic box submerged in a 37°C water bath and flushed for 2 h with a humidified mixture of 8% oxygen and 92% nitrogen, delivered at a rate of 2 L/min. After hypoxia-ischemia, the pups were returned to and kept with their dams (HI group). Hypoxia preconditioning (HP) was performed as described by Gidday et al. [13]. Six-day-old (P6) Sprague-Dawley rats were exposed to 8% oxygen for 3 h followed by a combined hypoxia-ischemia insult 1 day later, as described above (HP + HI group). Sham-operated controls received anesthesia and neck incision without carotid artery ligation and did not receive the hypoxia insult (sham group).

### 2.3. Hemispheric Weight Reduction

On day 14 after birth (P14), the pups were anaesthetized with isoflurane and decapitated and the brains were removed (*n* = 6 in each group). After removal of the brainstem and cerebellum, the forebrain was sectioned at the midline, and both hemispheric weights were determined. A previous study showed that the extent of unilateral reduction in hemispheric weight is highly correlated with biochemical, electrophysiological, and morphometric markers of tissue injury, in the same animal model [24]. Therefore, the percentage reduction in hemispheric weight was used as a measurement of cerebral injury in this study, calculated as (left hemisphere weight − right hemisphere weight)/left hemisphere weight.

### 2.4. Nissl and Immunohistochemistry Staining

Brains were collected 24 h after HI injury (P8) for immunohistochemistry assessment of glial activation and Nissl staining was performed for evaluation of structural and cellular damage (*n* = 5 in each group). The pups were anaesthetized with isoflurane and perfused transcardially with ice-cold saline followed by 4% paraformaldehyde in 0.1 M phosphate-buffered saline (PBS). Brains were then removed, postfixed overnight in 4% paraformaldehyde, and then transferred into a 30% sucrose solution for 72 h. At this time, brains were embedded and frozen in Neg-50 (Richard-Allan Scientific, Thermo Fisher Scientific, Kalamazoo, MI, USA) for storage at −80°C. Coronal sections were cut at 20 *μ*m on a Leica cryostat and collected serially onto gelatin-coated slides for storage at −80°C.

For Nissl staining, the brain sections were rinsed with PBS twice and then stained with 0.1% cresyl violet solution for 5 min. After rinsing quickly in distilled water, the sections were dehydrated in 100% alcohol for 10 min, cleared in xylene, and then mounted with permanent mounting medium.

Immunohistochemistry was performed to identify activated microglia and macrophages using the marker CD11b, and glial fibrillary acidic protein (GFAP) was used as a marker of activated astrocytes. Briefly, sections were thawed and treated with 3% hydrogen peroxide for 30 min. After blocking in 4% nonfat milk containing 0.4% Triton X-100 for 60 min at room temperature, the sections were incubated overnight at 4°C with primary antibodies against CD11b (1 : 200; Serotec, Oxford, UK) and GFAP (1 : 500; Lab Vision Corporation). Sections were then washed several times in PBS and incubated with a biotinylated secondary antibody and then treated using an avidin-biotin complex kit (ABC kit; Vector Laboratories. Burlingame, California). Finally, the labeling was detected by treatment with 0.01% hydrogen peroxide and 0.05% 3,3′-diaminobenzidine (DAB; Sigma, St. Louis, MO, USA). The DAB reaction was stopped by rinsing tissues in PBS. Labeled tissue sections were then mounted and analyzed under a bright-field microscope.

### 2.5. Hypoxia and Reoxygenation in a Mixed Primary Glial Culture and Microglial BV-2 Cell Line

Primary glial cultures were prepared according to a previous report [25]. In brief, mixed glial cultures were prepared using brains of 1-day-old pups from Sprague-Dawley rats. After anesthesia, the pups were decapitated, cerebral hemispheres were harvested, and the meninges removed. Minced pooled tissue was then digested with 0.25% trypsin, centrifuged, and dissociated into a single-cell suspension by gentle trituration. After filtration, the cells were seeded at a density of 1 × 10^7^ cells into 75 cm^2^ flasks in Dulbecco's modified Eagle's medium (DMEM; Gibco, Grand Island, NY, USA) containing 10% heat-inactivated fetal bovine serum (FBS; Gibco), 2 mM L-glutamine, 1 mM sodium pyruvate, 100 mM nonessential amino acids, 100 U/mL penicillin, and 100 mg/mL streptomycin. Cell cultures were maintained at 37°C in a humidified atmosphere of 5% CO_2_ and 95% air, and medium was replenished twice a week. These cells were used for experiments after reaching confluence (7-8 days).

The murine BV-2 cell line was cultured in DMEM with 10% FBS at 37°C in a humidified incubator in 5% CO_2_ and 95% air. Confluent cultures were passaged by trypsinization. All the cells were plated on 12-well plates at a density of 2.5 × 10^5^ cells/well for nitrite assays or on 6-well plates at a density of 5 × 10^5^ cells/well for reverse transcriptase-polymerase chain reaction (RT-PCR). The cells were then cultured for 2 days before experimental treatment.

Hypoxia/reoxygenation was performed as previously described with some modifications [25]. To generate the hypoxic condition, the cultures were gassed with 0.5% O_2_, 94.5% N_2_, and 5% CO_2_ (Ruskinn Hypoxic Gas Mixer, Model INVIVO2 200, serial number BBD902300), and control cultures were incubated under normoxic conditions for the same duration. After the indicated hypoxic period, reoxygenation was performed by transferring the cells into a regular normoxic incubator (95% air, 5% CO_2_), and cells were incubated for another 24 h for nitrite assays. For RT-PCR, total RNA was extracted from the cells after termination of hypoxia, using the same method outlined above.

### 2.6. Cell Viability and Nitrite Assays

Cell viability was assessed by the 3-(4,5-dimethylthiazol-2-yl)-5-(3-carboxymethoxyphenyl)-2-(4-sulfophenyl)-2H-tetrazolium (MTT) assay. Cells cultured in 12-well plates were exposed to hypoxia (0.5, 1, 2, and 4 h) or lipopolysaccharide (1 *μ*M). After incubation for 24 h, 20 *μ*L CellTiter 96 AQueous One Solution Reagent (Promega, Madison, WI, USA) was added to culture wells for 30 min. One hundred microliters of the culture medium from each well was transferred to an ELISA 96-well plate, and absorbance at 490 nm was measured with a 96-well plate reader. The absorbance at 490 nm is directly proportional to the number of living cells in culture.

Accumulation of nitrite in the medium was determined using a colorimetric assay with Griess reagent. Briefly, 100 *μ*L of culture supernatant reacted with an equal volume of Griess reagent (one part 0.1% naphthylethylenediamine and one part 1% sulfanilamide in 5% H_3_PO_4_) in 96-well cell culture plates for 10 min at room temperature in the dark. Nitrite concentrations were determined by using standard solutions of sodium nitrite prepared in cell culture medium. The absorbance at 550 nm was determined using a microplate reader (BioTek, Winooski, VT, USA).

### 2.7. Reverse Transcriptase-Polymerase Chain Reaction

Total RNA was extracted from brain tissue homogenates (*n* = 4 in each group) or cell culture (*n* = 5 in each group) using an RNeasy mini Kit (Qiagen, Valencia, CA, USA). Two micrograms of RNA was used for RT-PCR. The reaction mixture contained 1 *μ*g Oligo (dT)_15_ Primer, 0.02 mM deoxynucleotide triphosphate (dNTP), 40 U RNase Inhibitor, 100 U M-MLV Reverse Transcriptase, and 5x Reaction Buffer (Promega Corporation, Madison, WI, USA). PCR was performed using an initial step of denaturation (5 min at 94°C), 25 cycles of amplification (94°C for 30 s, 55°C for 30 s, and 72°C for 30 s), and an extension (72°C for 7 min). PCR products were analyzed on 1.5% agarose gels. The mRNA of glyceraldehyde 3-phosphate dehydrogenase (GAPDH) served as the internal control for sample loading and mRNA integrity. All of the mRNA levels were normalized to the level of GAPDH expression. The oligonucleotide primers are shown in Table 1.

### 2.8. Detection of Inflammatory Mediators by ELISA

Two major proinflammatory cytokines, tumor necrosis factor-alpha (TNF-*α*) and interleukin-1 beta (IL-1*β*), were detected using ELISA kits (R&D Systems, Minneapolis, MN, USA), according to the manufacturer's instructions. Briefly, pups were sacrificed by decapitation at 2, 6, 12, 24, and 72 h after HI (*n* = 4 for each time point per group) and protein samples of the ipsilateral hemispheric cortex from each pup were collected. All protein concentrations were determined by the Bradford method (Bio-Rad Bulletin 1177, Bio-Rad Laboratories, Hercules, CA, USA). Data were acquired using a 96-well plate reader (BioTek). The cytokine content is expressed as pg cytokines/mg protein.

### 2.9. Statistical Analysis

Data are presented as mean ± SEM. The comparisons for brain weight reduction were performed by one-way analysis of variance (ANOVA). The mRNA levels and protein concentrations at different time points were analyzed by two-way ANOVA to compare the HI and HP + HI groups. Individual groups were then compared using Bonferroni's post hoc tests, as appropriate. Statistical analyses were performed using Prism GraphPad v.5.0 (GraphPad Software, San Diego, CA, USA), with a significance level of *P* < 0.05.

## 3. Results

### 3.1. Hypoxia Preconditioning Is Neuroprotective and Attenuates Microglial and Astroglial Activation after a Hypoxia-Ischemia Insult in Neonatal Rats

**Figure 1:**
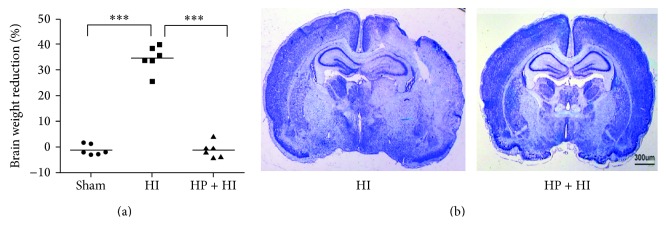
Hypoxia preconditioning exerts significant neuroprotection in neonatal hypoxia-ischemia brain injury. (a) The ratio of hemispheric brain weight reduction measured 7 days after hypoxia-ischemia injury was significantly reduced in the hypoxia preconditioning group (HP + HI), compared with the hypoxia-ischemia only (HI) group. (b) Nissl stained frozen sections showed extensive brain damage in the ipsilateral hemisphere 24 h after hypoxia-ischemia injury (HI, left panel), which was attenuated by hypoxia preconditioning (HP + HI, right panel).

The mean ratio of brain weight reduction of the right hemisphere, measured 7 days after right carotid artery ligation and exposure to 2 h of hypoxia in the HI group, was 34.6 ± 2.1%; this reduction was significantly greater than in the HP + HI group. The ratio of brain weight reduction was not statistically different between HP + HI and sham groups. The percentages for brain weight reduction are individually illustrated in Figure 1(a). Twenty-four hours after the hypoxia-ischemia insult, Nissl stained coronal sections at the level of the dorsal hippocampus demonstrated widespread injury in the right hemisphere of the HI group (Figure 1(b)). The numbers of normal-appearing neurons in neocortex, hippocampus, and striatum were also markedly decreased in the HI group. In contrast, brain weight reductions were significantly attenuated, and there was a marked increase in the number of normal-appearing neurons in rats that underwent 3 h of hypoxia preconditioning 24 h before hypoxia-ischemia injury (HP + HI group). The brain sections in the HP + HI group also showed minimal morphological damage. Hypoxia preconditioning in P7 rats provided nearly complete neuroprotection against hypoxia-ischemia injury, based on morphological indicators.

**Figure 2:**
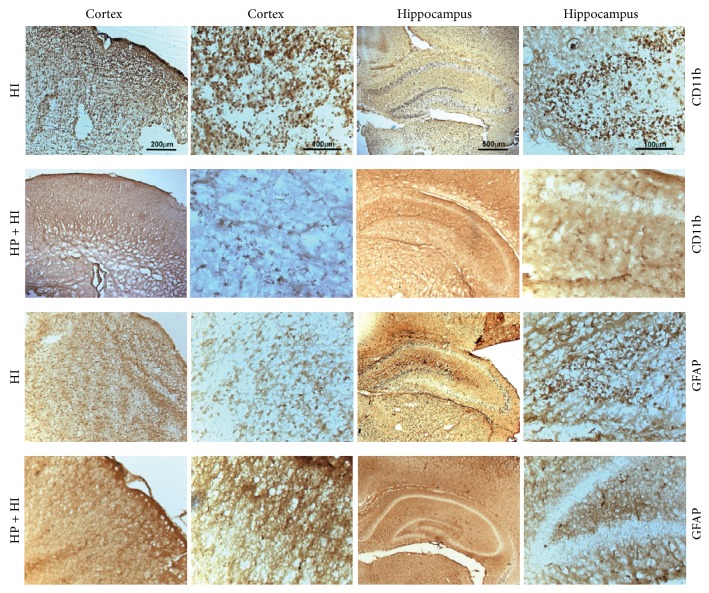
Hypoxia preconditioning inhibits microglial and astroglial activation in neonatal hypoxia-ischemia brain injury. Hypoxia-ischemia (HI) significantly induced microglial (first panel, CD11b) and astroglial activation (third panel, GFAP) in the cerebral cortex and hippocampus, which was correlated with neuronal damage (as shown in Figure 1). Hypoxia preconditioning (HP + HI) diminished the activation of microglial (second panel, CD11b) and astroglial cells (fourth panel, GFAP) after hypoxia-ischemia injury. Based on morphological observations, most microglial cells in the representative section were in an activated state in HI group, but a ramified form (resting state) in the HP + HI group.

To identify the effect of hypoxia preconditioning on glial response after hypoxia-ischemia injury, we used CD11b immunostaining to determine microglial activation and GFAP immunostaining to determine astrocytic activation (Figure 2). Twenty-four hours after hypoxia-ischemia injury in the HI group, numerous CD11b-positive microglia and GFAP-positive astroglia were observed in the ipsilateral hemisphere. These cells were scattered throughout the entire cortex and hippocampus. Most microglia were round-shaped with thick processes, and these cells were considered to be in an activated state. The reactive astroglia were also detected in the ipsilateral hemisphere and had a similar spatial distribution to the activated microglia. In the HP + HI group, the numbers of CD11b-positive microglia and GFAP-positive astroglia were much less than in the HI group. The morphology of microglia in the HP + HI group was also different to the HI group. The CD11b-positive cells in the ipsilateral hemisphere of rats from the HP + HI group (Figure 2, second lane) and in the contralateral hemispheres of the HI rats (not shown) appeared as ramified microglia, indicative of the resting state. These results demonstrate that the activation of microglia and astroglia following neonatal hypoxia-ischemia injury was attenuated by hypoxia preconditioning.

### 3.2. Hypoxia Preconditioning Suppresses Hypoxia-Ischemia Injury-Induced Inflammation in Neonatal Brain

**Figure 3:**
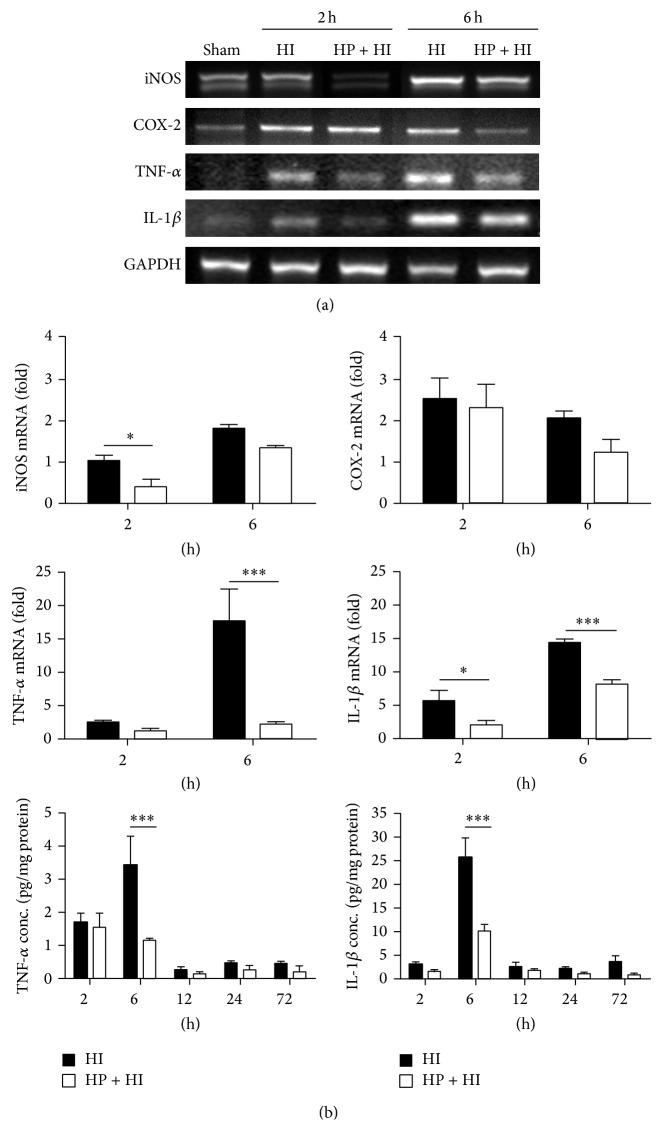
Hypoxia preconditioning reduces proinflammatory gene expression in neonatal hypoxia-ischemia brain injury. (a) The mRNA levels of inducible nitric oxide synthase (iNOS), cyclooxygenase-2 (COX-2), tumor necrosis factor-alpha (TNF-*α*), and interleukin-1 beta (IL-1*β*) in the ipsilateral cerebral cortex from neonatal rats were determined at 2 and 6 h after hypoxia-ischemia (HI) injury (*n* = 4 in each group). The gene expression levels were upregulated by HI injury, especially at 6 h after injury. Hypoxia preconditioning (HP + HI) was able to significantly suppress these responses. (b) The protein concentrations of TNF-*α* and IL-1*β* were determined by ELISA at different time points (*n* = 4 in each group). The suppression of inflammatory mediators in the HP + HI group was significant at 6 h after HI. ((a), (b)) Data represented as the mean ± standard error of the mean (SEM). ^*^ *P* < 0.05 compared with HI, ^***^ *P* < 0.001 compared with HI, and *P* = 0.08 for COX-2 mRNA level at 6 h for HP + HI group compared with HI group.

We next examined the effect of hypoxia preconditioning on the inflammatory response induced by hypoxia-ischemia injury. Comparing the mRNA levels of the right cortex in the HP + HI group with the HI group, inducible NOS (iNOS) and IL-1*β* upregulation were significantly reduced as early as 2 h after the hypoxia-ischemia insult (Figure 3(a)). Six hours after the hypoxia-ischemia insult, mRNA expression of all inflammatory mediators was markedly increased in the HI group. Hypoxia preconditioning significantly suppressed upregulation of the cytokine genes, TNF-*α* and IL-1*β*, at 6 h. Hypoxia preconditioning also suppressed COX-2 upregulation 6 h after hypoxia-ischemia injury in the HP + HI group (but this only reached marginal significance; *P* = 0.08). We thus extracted total protein from the right cortex and further evaluated TNF-*α* and IL-1*β* protein expression by ELISA at different time points (Figure 3(b)). The levels of TNF-*α* were very low in the sham group (0.08 ± 0.02 pg/mg protein) and were increased markedly at 2 h after the hypoxia-ischemia insult (1.65 ± 0.32 pg/mg protein, HI group). These levels peaked at 6 h (3.42 ± 0.88 pg/mg protein, HI group) and were decreased after 12 h. Hypoxia preconditioning resulted in a significant reduction of the TNF-*α* level at 6 h (1.16 ± 0.04 pg/mg protein, HP + HI group). The level of IL-1*β* was also very low at baseline (0.71 ± 0.1 pg/mg protein, sham group), slightly increased 2 h after the hypoxia-ischemia insult (3.15 ± 0.52 pg/mg protein, HI group), and markedly elevated 6 h after the hypoxia-ischemia insult (25.97 ± 3.89 pg/mg protein, HI group). Hypoxia preconditioning before the hypoxia-ischemia insult markedly decreased the IL-1*β* levels at 6 h (10.19 ± 1.47 pg/mg protein, HP + HI group). Therefore, hypoxia preconditioning was able to suppress the hypoxia-ischemia injury-induced inflammatory response in neonatal rat brain.

### 3.3. Hypoxia Preconditioning Suppresses Hypoxia-Induced Glial Cell Activation* In Vitro*

**Figure 4:**
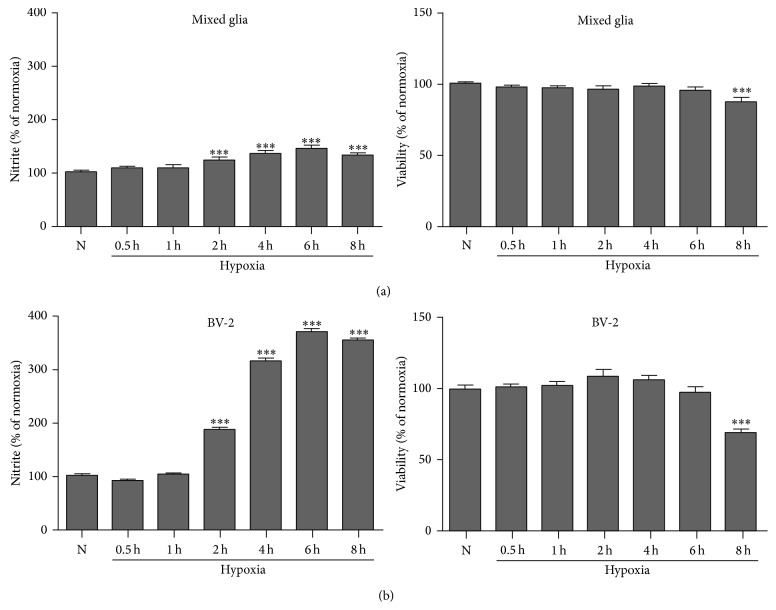
Hypoxia insult induces glial activation in cultured glial cells without reducing cell viability. Nitrite production and cell viability were determined in a primary mixed glial culture (a) and in the BV-2 microglial cell line (b) after exposure to hypoxia, at various time intervals (as indicated). Hypoxia durations up to 6 h increased nitrite production in a time-dependent manner without reducing cell viability. Hypoxia exposure for 8 h was able to induce a low level of cell death. The data represent the mean ± standard error of the mean (SEM), based on five independent experiments in each group. ((a), (b)) ^***^ *P* < 0.001 compared with HI group.

To further clarify the role of glial cells in the anti-inflammatory mechanisms of hypoxia preconditioning, we next examined the glial response to hypoxia preconditioning at the cellular level. Firstly, nitrite production and cell viability after hypoxia exposure for different time durations were determined in a primary mixed glial culture and in the microglial cell line, BV-2 (Figure 4). Exposure to hypoxic conditions for less than 1 h did not increase nitrite production in either the primary mixed glial culture or the BV-2 cell line. However, nitrite production increased as hypoxia duration increased, for exposure times exceeding 1 h. Cell viability was not significantly changed, except for 8 h of hypoxia.

**Figure 5:**
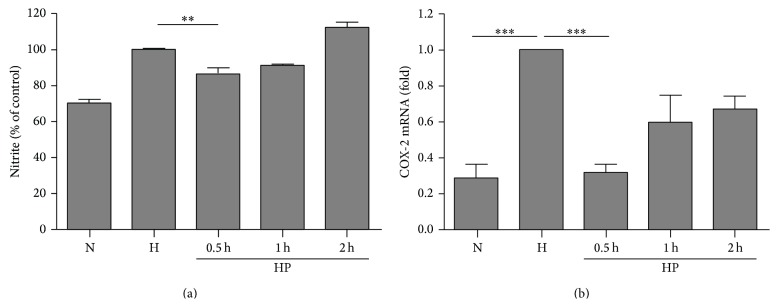
Hypoxia preconditioning suppresses prolonged hypoxia-induced inflammation in primary mixed glial culture. (a) Brief hypoxia exposure for 0.5 h before prolonged hypoxia damage (4 h) significantly reduced the production of nitrite. ^**^ *P* < 0.01 compared with hypoxia control. (b) mRNA expression of cyclooxygenase-2 (COX-2) was also significantly suppressed by brief hypoxic preconditioning for 0.5 h. ^***^ *P* < 0.001 compared with normoxia and hypoxia control. ((a), (b)) The data represent the mean ± standard error of the mean (SEM), based on five independent experiments in each group.

**Figure 6:**
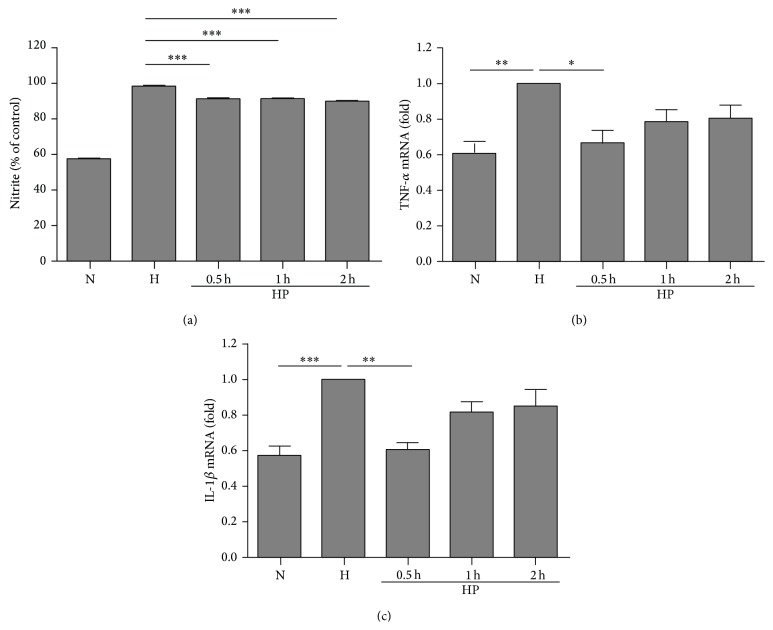
Hypoxia preconditioning suppresses prolonged hypoxia-induced inflammation in the BV-2 microglial cell line. (a) Brief hypoxia exposure (0.5, 1, and 2 h) before prolonged hypoxia damage (4 h) significantly reduced the production of nitrite. ^***^ *P* < 0.001 compared with hypoxia control. (b) mRNA expression of tumor necrosis factor-alpha (TNF-*α*) was suppressed by brief hypoxia preconditioning. ^**^ *P* < 0.01 compared with normoxia control and ^*^ *P* < 0.05 compared with hypoxia control. (c) mRNA expression of interleukin-1 beta (IL-1*β*) was also suppressed by brief hypoxia preconditioning. ^***^ *P* < 0.001 compared with normoxia control and ^**^ *P* < 0.01 compared with hypoxia control. ((a), (b), (c)) The data represent the mean ± standard error of the mean (SEM), based on five independent experiments in each group.

We then used mixed primary glial cultures to investigate the effect of hypoxia preconditioning. The cells were preexposed to hypoxia for 0.5, 1, or 2 h and then returned to the normoxia incubator. Twenty-four hours later, these cells were incubated in a hypoxia chamber again for 4 h, which mimics the* in vivo* model of hypoxia preconditioning. The production of nitrite after reoxygenation was decreased significantly by hypoxia preconditioning for 0.5 h, but not for 1 or 2 h (Figure 5(a)), and cell viability stayed the same. The expression of proinflammatory genes was also evaluated by RT-PCR (Figure 5(b)). Hypoxia preconditioning for 0.5 h significantly suppressed the expression of COX-2 genes. These results indicate that hypoxia preconditioning for 0.5 h exerts the most pronounced effect on suppression of inflammatory mediators in primary mixed glial cultures. To examine the response of isolated microglia, the same experimental protocol was also applied in the BV-2 microglial cell line. The levels of nitrite were decreased significantly by 0.5–2 h hypoxia preconditioning (Figure 6(a)). The expression levels of TNF-*α* (Figure 6(b)) and IL-1*β* (Figure 6(c)) were suppressed by 0.5 h hypoxia preconditioning in the BV-2 cell line.

## 4. Discussion 

There is emerging evidence indicating that inflammatory processes play a pivotal role in the pathogenesis of neonatal brain injury [5, 9, 26]. In the present study, we found that the inflammatory cascade was initiated with the release of proinflammatory mediators within hours after exposure to a hypoxia-ischemia insult. The expression of proinflammatory genes increased as early as 2 hours after hypoxia-ischemia in the neonatal brain. The expression levels of TNF-*α* and IL-1*β* were even more significant 6 hours after the hypoxia-ischemia insult. The inflammatory events were accompanied by activation of resident glial cells. Twenty-four hours after the hypoxia-ischemia insult, extensive activation and proliferation of microglial and astroglial cells were noted in the cortex and hippocampus, with associated tissue damage. The astrocytes were hypertrophic and the microglial cells also appeared in their activated state, with an enlarged cell body and shorter but thicker cellular processes. Activation of glial cells enhances the inflammatory process and further exacerbates brain damage.

Our finding in the present study also complements the previous inference that 3 hours of hypoxia exposure, 24 hours before the hypoxia-ischemia insult, markedly reduces neonatal brain injury [13, 18, 27]. In our study, hypoxia preconditioning provides robust endogenous neuroprotection. Compared to the nonischemic left cerebral hemisphere, the brain weight reduction for the injured right hemisphere was 34.6%. However, hypoxia preconditioning 1 day before hypoxia-ischemia injury completely rescued right hemispheric structural damage, as illustrated in Figure 1. The left brain hemisphere in the HI group was exposed to only 2 hours of hypoxic gas without disruption of carotid artery blood flow. Based on the results of Nissl staining in the left hemisphere of the HI group, which revealed normal structure and cellularity, it could be argued that hypoxic preconditioning* per se* did not cause structural damage in normal rats, which is compatible with evidence from previous studies [13, 23]. Since the structural neuroprotective effect of hypoxia preconditioning is quite striking, further identification of the underlying mechanism may have an important role in the development of therapeutic strategies. Several pathways have been linked with hypoxia preconditioning in neurons [15, 21], but only a few studies assessed the role of glial cells [7, 28]. Our study demonstrates that hypoxia preconditioning attenuates activation and infiltration of microglial and astroglial cells after hypoxia-ischemia in neonatal brain. Most microglial cells in the ipsilateral cortex and hippocampus remained in the resting form in the hypoxia preconditioning group.

Hypoxia-ischemia-induced expression for iNOS, COX-2, TNF-*α*, and IL-1*β* was also significantly suppressed after hypoxia preconditioning treatment. The results suggest that hypoxia preconditioning also exerted anti-inflammatory effects in neonates that were subsequently exposed to a hypoxia-ischemia insult, through inhibition of glial activation. Whether all types of cerebral preconditioning share the same molecular mechanism is not yet clear and will need further investigation [29]. Rosenzweig et al. [30] demonstrated that LPS preconditioning suppressed neutrophil infiltration into the brain and microglial/macrophage activation in the ischemic hemisphere. The expression of TNF-*α* and production of reactive oxygen species were also reduced [31]. Similar to ischemic and LPS preconditioning, we have also demonstrated in this study that hypoxia preconditioning was able to suppress the cellular inflammatory response after exposure to global cerebral insults. Diminished activation of glial cell responses, which ordinarily exacerbates ischemic injury, may contribute to the neuroprotection conferred by hypoxia preconditioning.

It may be argued that a diminished inflammatory reaction arises from a reduction in neuronal damage and is not a direct cellular response. We thus developed an* in vitro* model of hypoxia preconditioning to assess the cellular response. In a mixed glial culture, a prior exposure to brief hypoxia 24 hours before the prolonged hypoxia insult significantly reduced the production of inflammatory mediators (such as nitrite) and the expression of the inflammatory related gene, COX-2. Our results also showed that a longer period of hypoxia preconditioning had less of an anti-inflammatory effect, which implies the importance of selecting the appropriate duration for hypoxia preconditioning. The induction of proinflammatory cytokine genes, including TNF-*α* and IL-1*β*, was not as prominent as for the* in vivo* model. The difference in cytokine response may be due to the recruitment of circulating white blood cells. In the animal model, hypoxia-ischemia will induce migration and infiltration of circulating macrophages and monocytes, further increasing proinflammatory cytokine release [32]. The major cell type in mixed glial culture is astroglia, which might have a lower production of cytokines. Thus, we further evaluated the microglial response to hypoxia preconditioning using the microglial cell line, BV-2. The production of nitrite and expression of TNF-*α* and IL-1*β* were all significantly increased after hypoxia exposure for 4 hours, and hypoxia preconditioning for the optimal duration (0.5 h) was able to attenuate the activation effects. Again, longer durations of hypoxia preconditioning decreased the anti-inflammation effects, as in primary mixed glia cultures.

Glial cells, in particular astrocytes, are usually viewed as supporters of neuronal function. However, numerous studies are increasingly demonstrating the important role of glial cells in preserving brain function under physiological and pathological conditions [33]. The generation of anti-inflammatory cytokines and trophic factors by preconditioned astrocytes might also contribute to neuroprotection [34]. The suppression of glial cell activation and subsequent inflammatory reactions, as shown in the present study, might contribute to the cerebral protective effects of hypoxia preconditioning.

The results of the present study can also be extrapolated to other mechanisms of neonatal brain injury. An increasing body of evidence has demonstrated a link between inflammation and long-term brain dysfunction. A recent meta-analysis of 26 articles has shown a positive association between infection and cerebral palsy, in both preterm and full-term infants [35]. In addition, the expression levels of inflammatory cytokines and chemokines in amniotic fluids and cord blood have been shown to correlate with neonatal brain injury and later developmental disability [36, 37]. In preventing these consequences, induction of an endogenous anti-inflammatory response by hypoxia preconditioning may be beneficial to protect neurons from brain damage.

## 5. Conclusions

In conclusion, hypoxic preconditioning induced significant neuroprotection against neonatal hypoxia-ischemia brain insults and suppressed astroglial and microglial activation in the ischemic cortex and hippocampus. Pretreatment with sublethal exposure to hypoxia before prolonged hypoxia injury prevented the cellular inflammatory response in the primary glial culture and microglial cell line BV-2. These results further address the importance of anti-inflammatory strategies in preventing neonatal brain injury.

## Figures and Tables

**Table 1:** List of primers used for RT-PCR.

Gene name	Accession number	Sequence	Position	Product length
iNOS	NM_010927.2	F 5′-CCTTGTTCAGCTACGCCTTC-3′	1841–1860	573 bp
		R 5′-GGCTGGACTTTTCACTCTGC-3′	2413–2394	

COX-2	NM_011198.3	F 5′-TGATGACTGCCCAACTCCCATG-3′	527–547	723 bp
		R 5′-AATGTTGAAGGTGTCCGGCAGC-3′	1249–1228	

TNF-*α*	NM_013693.2	F 5′-TCAGCCTCTTCTCATTCCTGC-3′	254–274	203 bp
		R 5′-TTGGTGGTTTGCTACGACGTG-3′	456–436	

IL-1*β*	NM_008361.3	F 5′-CTCCATGAGCTTTGTACAAGG-3′	545–565	245 bp
		R 5′-TGCTGATGTACCAGTTGGGG-3′	789–770	

GAPDH	XM_001478528.1	F 5′-ACCACAGTCCATGCCATCAC-3′	579–598	452 bp
		R 5′-TCCACCACCCTGTTGCTGTA-3′	1030–1011	
